# Study of Menstrual Hygiene Practices Among Adolescent Girls in a Tribal Area of Central India

**DOI:** 10.7759/cureus.30247

**Published:** 2022-10-13

**Authors:** Sonali K Borkar, Avinash Borkar, Mohammed K Shaikh, Harshal Mendhe, Ranjit Ambad, Abhishek Joshi

**Affiliations:** 1 Community Medicine, Datta Meghe Medical College, Datta Meghe Institute of Medical Sciences, Nagpur, IND; 2 Biochemistry, Datta Meghe Medical College, Datta Meghe Institute of Medical Sciences, Nagpur, IND; 3 Community Medicine, Jawaharlal Nehru Medical College, Datta Meghe Institute of Medical Sciences, Wardha, IND

**Keywords:** tribal residential school, restrictions, sanitary pads, tribal adolescent girls, menstrual hygiene

## Abstract

Introduction

Menstruation is an essential phenomenon in an adolescent girl's life. In India, females who are menstruating are considered impure and teenage girls are not allowed to undertake home chores or engage in religious or cultural events during their period. Up-to-date knowledge about menstruation, beginning in early adolescence, would improve safe practices and relieve the distress of millions of women.

Material and methods

A cross-sectional study was conducted among adolescent girls (10-19 years) residing in a tribal area of Nagpur District from January to March 2022. Three tribal residential schools (Ashram Shala) were selected from a total of six in Hingana Taluka of Nagpur District, using simple random sampling to fulfill the required sample size of 272. Demographic details, age at menarche, awareness of menstruation, sources of information about menstruation, menstrual hygiene practices, and restrictions observed during menstruation were assessed.

Results

The average age at menarche was 13.04+0.96 years (range 11-16 years). Only 45.17% of girls were aware of the menarche and menstrual cycle before its onset. The duration of the menstrual cycle ranged from 21-35 days in most of the girls (90.69%), and it was regular in 85.86% of girls. Duration of bleeding was two to six days for the majority of the girls (87.93%). Around 73.79% of girls were using sanitary pads, while 26.21% of girls were using clothes. The most important restrictions imposed on the girls during menstruation were not being allowed to attend religious functions (97.93%), followed by not being allowed to attend classes (65.86%).

The use of sanitary pads was significantly more in late adolescent girls than in early adolescent girls (ꭓ^2^=14.97, p=0.0001), girls who have literate mothers than girls with illiterate mothers (ꭓ^2^=5.17, p=0.02), and girls belonging to higher socioeconomic classes (class I, II, III) than lower ones (class IV, V) (ꭓ^2^=44.23, p<0.0001).

Conclusion

The tribal adolescent girls still don't exercise proper hygiene throughout their periods. During menstruation, the majority of girls are still subjected to various restrictions.

## Introduction

Adolescence is a unique time in a woman's life that represents the shift from youth to adulthood. Menstruation is an essential phenomenon in an adolescent girl's life. She undergoes various physiological and psychological changes during this phase of life. Menstruation is considered unclean in India, and teenage girls are not allowed to undertake home chores or engage in religious or cultural events during their period [1]. The problem of improper menstrual hygiene is scarcely acknowledged in developing countries like India. Lack of menstrual hygiene is connected with negative effects such as infections of the reproductive and urinary tract, which may lead to future infertility and birth complications [2]. Proper knowledge about menstrual hygiene and its application can improve adolescent girls' reproductive health to a great extent.

Girls' and women's health, education, and integrity are all dependent on good menstrual hygiene [3]. Menstrual hygiene is influenced by a family's educational, social, and cultural status. There is still a pressing need to openly confront this significant issue because it has gone unattended for so long. Since the majority of girls in various regions of the country, particularly in rural, urban slums, and tribal areas, are neither prepared for nor aware of menstruation - in terms of knowledge, attitudes, and skills - they experience various challenges and difficulties at home, in schools, and at work. Therefore, up-to-date knowledge about menstruation, beginning in early adolescence, would improve safe practices and relieve the distress of millions of women [2].

According to the National Family Health Survey 5 (NFHS-5), women between the ages of 15 and 24 years who safeguard themselves throughout their menstrual period with hygienic ways are 77.3% in India (urban 89.4%, rural 72.3%) [4]. Various studies have been carried out in Maharashtra [5,6,7], but recent data is not available for the tribal population in Central India; so the current study was carried out with the objective of determining the awareness regarding menstruation and sources of information before the onset of menarche in tribal adolescent girls. The study also seeks to analyze tribal adolescent girls' existing menstrual hygiene practices and to evaluate the association of practices for menstrual hygiene with the age and socioeconomic status of tribal adolescent girls and the educational and working status of their mothers.

## Materials and methods

Study design and setting

A cross-sectional observational study was carried out in a rural area under Datta Meghe Medical College, Wanadongari, Nagpur. The study was conducted from January to March 2022 in tribal schools (Ashram Shala) of Hingana taluka in Nagpur district, which is the catering area of the medical college and hospital.

Sample size calculation and sampling technique

Using the formula to calculate sample size, n=z2p(1-p)/e2, where z is the standard normal deviate at 95% CI, 'p' is the prevalence of sanitary pad usage, and 'e' is the allowable error, the sample size was determined. The value for p was taken from previously conducted studies as 77 [4]. Allowable error e was taken as 5%. The required sample size was calculated using the above parameters came out to be 272.

There are a total of six tribal residential schools in Hingana Taluka, with 1579 students studying from first to 10th standard. To fulfill the required sample size for the study, three of these six schools were selected by simple random sampling using the lottery method. There were 878 students in these schools, with 345 adolescent girls.

Inclusion and exclusion criteria

All adolescent girls (10-19 years) residing in the tribal residential school were included in the study. There were 345 female students fulfilling this criterion. Of these, 55 girls who had not attained menarche were excluded from the study. Thus, a total of 290 girls were included in the study.

Prior permission was taken from the Tribal Health Department and school authorities. The study's objective was outlined to the class teachers of the secondary and higher secondary classes and the parents of the girls before the start of the study. Ethical permission was obtained from the Institutional Ethical Committee.

Data collection

The study was carried out from January to March 2022. A total of 290 girls were interviewed. A rapport was developed with each student, and a personal interview was conducted in a separate room by a female investigator. A pre-tested structured questionnaire designed in the local language was used to collect data. The parameters in this questionnaire included demographic details, age at menarche, awareness of menstruation, and sources of information about menstruation. Socioeconomic status was assessed using the Modified BG Prasad Scale [8]. Menstrual hygiene practices and restrictions observed during menstruation were assessed during the interview. The time required for each interview was around 20-30 minutes. After collecting data, any queries of students regarding menstrual hygiene were solved.

Statistical analysis

Data was collected and entered in a Microsoft Excel (Microsoft, Redmond, Washington) worksheet for the purpose of analysis and calculation of mean, proportions, and percentages. The statistical test of significance used was the Chi-square test, and statistical significance was decided at the level of p-value<0.05.

## Results

Table 1 shows the sociodemographic characteristics of the tribal adolescent girls. Out of 290 girls, 121 (41.72%) were in early adolescence (10-14 years), and 169 (58.28%) were in late adolescence (15-19 years). The majority of them were Hindu 224 (77.24%), followed by Buddha 43 (14.83%) and Muslim 17 (5.86%). Regarding the mother's education, 128 (44.14%) were illiterate, 94 (32.41%) were educated up to the seventh standard, 66 (22.76%) had studied up to the 12th standard, and only two (0.69%) were graduates. One hundred and twenty-two (42.07%) mothers of the girls were housewives, whereas 168 (57.93%) were working women. The majority of girls belonged to lower-middle (81, 27.93%) and lower (93, 32.07%) socioeconomic status.

**Table 1:** Sociodemographic characteristics of the tribal adolescent girls (n=290)

Sociodemographic characteristics	No.	%
Age group		
10- 14 years	121	41.72
15-19 years	169	58.28
Religion		
Hindu	224	77.24
Buddha	43	14.83
Muslim	17	5.86
Christian	6	2.07
Education of mother		
Illiterate	128	44.14
Up to 7^th^ standard	94	32.41
8^th^ to 12^th^ Standard	66	22.76
Graduate	2	0.69
Working status of the mother		
Housewife	122	42.07
Working	168	57.93
Socio-economic status (Modified BG Prasad Scale)		
Upper (I)	23	7.93
Upper Middle (II)	42	14.48
Middle (III)	51	17.59
Lower Middle (IV)	81	27.93
Lower (V)	93	32.07

Table 2 shows the menstrual profile of the tribal adolescent girls. Regarding the menstrual profile of the girls, the average age at menarche was observed to be 13.04 + 0.96 years (range 11 -16 years). Most of the girls (206, 71.03%) attained menarche up to the age of 13 years. The duration of the menstrual cycle was found to be 21-35 days for most of the girls (263, 90.69%). The duration of bleeding was two to six days for the majority of the girls (255, 87.93%). The menstrual cycle was regular for most girls (249, 85.86%).

**Table 2:** Menstrual profile of the tribal adolescent girls (n=290)

Menstrual profile	No.	%
Age at menarche		
11 years	17	5.86
12 years	57	19.66
13 years	132	45.52
14 years	69	23.79
15 years	12	4.14
16 years	3	1.03
Duration of the menstrual cycle		
Up to 21 days	6	2.07
21-35 days	263	90.69
More than 35 days	21	7.24
Duration of bleeding		
< 2 days	12	4.14
2-6 days	255	87.93
> 6 days	23	7.93
Menstrual cycle pattern		
Regular	249	85.86
Irregular	41	14.14
Awareness about menstruation before the onset of menarche		
Yes	131	45.17
No	159	54.83

One hundred and thirty-one (45.17%) girls were aware of menarche and menstruation before its onset. Common sources of information were mother (71, 54.20%), sister (31, 23.66%), and social media (14, 10.69%); others being friends, teachers, and other relatives.

Table 3 shows adolescent girls' practices during menstruation. Regarding practices of the adolescent girls, 214 (73.79%) used sanitary pads, and 76 (26.21%) girls used clothes. Thirty-eight (13.10%) girls changed the absorbent material once a day, and 157 (54.14%) girls changed it twice daily. Seventy-three (25.17%) girls threw the absorbent material in the dustbin, but 141 (48.62%) threw it anywhere indiscriminately. In washing and reusing the absorbent material, 52 (68.42%) girls dried it inside the house and 24 (31.58%) outside the house. The hygienic practice of washing external genitalia every time while changing the pad was followed by only 78 (26.90%) girls. Only 113 (38.97%) girls cleaned their external genitalia with soap and water.

**Table 3:** Adolescent girls' practices during menstruation

Variable	No.	%
Material (absorbent) used		
Sanitary pad	214	73.79
Clothes	76	26.21
Frequency of change of absorbent		
Once daily	38	13.10
Twice daily	157	54.14
More frequently	95	32.76
Method of disposal of absorbent		
Washed and reused	57	19.66
Collected and burnt	19	6.55
Thrown in dustbin	73	25.17
Thrown anywhere	141	48.62
Place of drying absorbent (n=76)		
Inside house	52	68.42
Outside house	24	31.58
The practice of cleaning external genitalia		
Every time while changing the absorbent	78	26.90
While bathing only	212	73.10
Washing external genitalia with		
Only water	177	61.03
Soap and water	113	38.97

Table 4 shows restrictions imposed on the girls during menstruation. The most important restrictions imposed on the girls during menstruation were not being allowed to attend religious functions (284, 97.93%), followed by not being allowed to attend classes (191, 65.86%). Ninety-three (32.07%) girls also responded as isolation in a separate place, not going out or playing (73,25.17%), and avoiding eating certain foods (55, 18.97%) as restrictions imposed on them.

**Table 4:** Restrictions imposed during menstruation * Multiple responses

Restrictions *	No.	%
Not allowed to attend religious functions	284	97.93
Not allowed to attend classes	191	65.86
Isolation in a separate place	93	32.07
Not going out/playing	73	25.17
Avoiding eating certain foods	55	18.97

Table 5 shows the association between sociodemographic traits and the use of sanitary pads for menstruation. The use of sanitary pads was more in late adolescent than in early adolescent girls (ꭓ^2^=14.97, p=0.0001), girls who have literate mothers than girls with illiterate mothers (ꭓ^2^=5.17, p=0.02), and girls belonging to higher socioeconomic classes (class I, II, III) than lower ones (class IV, V) (ꭓ^2^=44.23, p<0.0001). This difference was discovered to have statistical significance. Girls who have working mothers used sanitary pads more frequently than girls whose mothers were housewives (ꭓ^2^=3.55, p=0.059), but this difference was statistically not significant.

**Table 5:** Sociodemographic variables and use of sanitary pads during menstruation

Variable	Sanitary pad usage		Chi-square value	p-value
	Yes (n=214)	No (n=76)		
Age in years				
10-14 years	75	46	14.97	0.0001
15-19 years	139	30		
Mother’s education				
Illiterate	86	42	5.17	0.02
Literate	128	34		
Mother’s occupation				
Working	117	51	3.55	0.059
Housewife	97	25		
Socioeconomic status				
Class I, II, III	110	6	44.23	0.00001
Class IV, V	104	70		

## Discussion

In the present study, the average age at menarche was found to be 13.04 ± 0.96 years (range 11-16 years) which is similar to studies conducted in Andhra Pradesh in 2016 and Madhya Pradesh in 2021 [3,9]. In a study conducted in Karnataka in 2020, the average age at menarche was 12-14 years which is comparable with the current research [10].

Awareness about menstruation before the onset of menarche was found to be 45% in the present study, comparable to a study conducted in Madhya Pradesh in 2021 (39%) [9]. In contrast, awareness was very low (12%) among rural school girls in a study conducted in Karnataka in 2022 [11].

Sanitary pad usage during menstruation was 74% in the present study. A study conducted in Karnataka in 2020 showed that 70% of adolescent girls used sanitary napkins when they were menstruating, and a study in Andhra Pradesh in 2016 and a study in Uttarakhand in 2021 showed it to be 78.5% and 79.5%, respectively, whereas a study in Jodhpur, Rajasthan in 2020 showed sanitary pad usage as 85% [3,10,12,13]. In contrast to these findings, sanitary pad usage during menstruation was found to be only 31% in a study in Madhya Pradesh in 2021 [9].

In a study by Kathuria et al. in 2022, regional variations in India regarding sanitary pad usage showed that it was highest in the Southern region (80%) and lowest in the Eastern region (44%) [14].

The duration of menstrual cycles was found to be 21-35 days for the majority of study subjects (90.69%) in the present study, which was similar to a study conducted in Karnataka in 2020 [10].

The correct way to dispose of absorbent materials used in periods, i.e., throwing it in a dustbin, was followed by only 25% of adolescent girls in the current study. Better results (around 60%) were obtained in the studies conducted in Karnataka in 2020 and Uttarakhand in 2021 [10,12]. This variation could be explained by the fact that tribal girls in the current study were compared to urban girls in the reference studies.

The most common restrictions imposed on adolescent girls during menstruation were not being allowed to attend religious functions (98%), not being allowed to go to school (66%), and isolation in a separate room or place (32%) in the current study. Similarly, in a study conducted in Delhi in 2021, restrictions were religious restrictions (94%), followed by routine activity restriction (69%), and restriction of academic activities (60%) [15]. Both of these studies, which targeted the indigenous population, had equivalent findings. The proportion of restrictions imposed varied in urban and rural girls (35-41%), as seen in a study conducted in Karnataka in 2018 [11].

In the present study, a significant association was found between sanitary pad usage and age of the adolescent girls (early and late adolescence), mother's education (illiterate and literate), and higher socioeconomic status (class I, II, III) and lower socioeconomic status (class IV, V). The health of future generations is significantly influenced by the role of mothers. The majority of girls learn about menstrual health through their mothers. In a study conducted in Uttar Pradesh and Bihar in 2021, a significant association was found between sanitary pad usage and mothers' education and wealth index, similar to the current study [16]. In contrast to these findings, in a study conducted in Assam in 2021, no significant association was found between sanitary pad usage and te age of participants, education of the mother, and socioeconomic status [17].

The limitation of the study is that the current study is being conducted in a tribal setting, and the findings cannot be generalized to the rural or urban population.

## Conclusions

The current study observed that the tribal adolescent girls still don't exercise proper hygiene throughout their periods. During menstruation, the majority of girls are still subjected to various restrictions. It was also observed that girls hesitate when discussing sensitive topics like menstrual hygiene; thus, it is important to create a healthy environment where everyone can talk honestly about their personal experiences with this type of delicate subject. There is a dire need to create awareness and promote cleanliness habits during periods in tribal adolescent girls. Teachers of residential schools can play a crucial role in this regard.
